# Oxidative stress impact on growth hormone secretion in the eye

**DOI:** 10.3325/cmj.2015.56.326

**Published:** 2015-08

**Authors:** Borna Šarić, Vlatka Brzović Šarić, Monika Barberić, Jurica Predović, Vlatko Rumenjak, Branimir Cerovski

**Affiliations:** 1 Ophthalmology Clinic, University Hospital Sveti Duh, Zagreb, Croatia; 2 Institute for Medical Laboratory Diagnostics, University Hospital Sveti Duh, Zagreb, Croatia; 3 University Hospital Center Zagreb, Zagreb, Croatia

## Abstract

**Aim:**

To evaluate the influence of oxidative stress on extrapituitary growth hormone (GH) secretion in the eye and to analyze the interdependence between eye and serum GH levels under normal and hypoxic conditions.

**Methods:**

Pars plana vitrectomy (PPV) was performed in 32 patients with developed proliferative diabetic retinopathy (PDR) and 49 non-diabetic controls, both of whom required this procedure as part of their regular treatment in the period from April 2013 to December 2014. During PPV, vitreous samples were taken and blood was simultaneously collected from the cubital vein. GH levels in serum and vitreous samples were measured by electrochemical luminescence assay. Oxidative stress was measured by enzyme-linked immunosorbent assay of advanced oxidation protein products (AOPP) and lipid hydroperoxide (LPO) in serum and vitreous.

**Results:**

Serum AOPP levels were significantly higher than vitreous levels in both groups (*P* < 0.001 for each group) and LPO levels were significantly higher only in PDR group (*P* < 0.001). There was a significant positive correlation between serum and vitreous LPO levels in PDR group (r = 0.909; *P* < 0.001). Serum GH levels were significantly higher than vitreous levels in both groups (*P* < 0.001 for each group). Serum GH levels were significantly higher in PDR group than in controls (*P* = 0.012). Vitreous GH values were slightly higher in PDR group, but the difference was not significant.

**Conclusion:**

Our study confirms that GH production in the eye is autonomous and independent of oxidative stress or pituitary GH influence.

The latest research has indicated that the secretion of growth hormone (GH) in human retinal ganglion cells (RGC) is maintained throughout the postembryonic life, and has a neuroprotective role (1-8). Serum human GH levels are elevated in diabetes and their possible correlation with etiopathogenesis of diabetic retinopathy (DR) has been investigated, but not confirmed (9-12). Diabetes is considered to be a primarily metabolic disorder that, besides leading to other complications, affects small and large blood vessels, thus causing hypoxia and oxidative stress in different tissues and organs. Therefore, DR represents an ideal model for exploring extrapituitary GH release, as well as various aspects of serum GH production (13). However, we still do not know the exact mechanism of how serum human GH interferes with the development of DR. The key assumption is that reduced amounts of insulin in the portal circulation reduce insulin growth factor 1 (IGF1) production in the liver with the final lack of feedback inhibition of increased GH secretion (12,13). All this takes place in conditions of hyperglycemia that is being suppressed by introduction of additional amounts of insulin, which provides the necessary condition for increased portal IGF1 production. In this stage of the disease, circulating values of both GH and IGF1 are elevated in serum (11,13,14) and this stage is usually associated with the appearance of DR. It has been proven that IGF1 exerts a promotional effect on neovascularization and proliferative changes in the eye, but it is unclear whether the increased levels of IGF1 measured in diabetic vitreous are derived from the passage of serum IGF1 through the blood retinal barrier (BRB) or it is autonomous growth factor created within the eye (15,16).

There are controversial findings on the effects of iatrogenic GH medical suppression in serum on DR. Some earlier studies, primarily on animal models, have shown that suppression of serum GH decreases the synthesis of IGF1 and thus slows down DR progression (17,18). Recent studies on a large number of human participants have shown that suppression of serum GH, despite the accompanying reduction in serum IGF1 levels, does not affect the stage of DR, which implies the existence of an autonomous IGF1 production in the eye (16,19).

It should be noted that insulin is significant IGF 1 secretion promoter just like GH, and that patients with type I and some with type II diabetes receive insulin therapy as their standard treatment procedure. Nevertheless, in such conditions insulin increasingly penetrates the BRB and additionally stimulates autonomous creation of IGF1 in the eye (20-23). In normal conditions, this barrier is impermeable to GH and IGF1, but animal model studies showed that in conditions of hypoxia and oxidative stress it became permeable to both molecules (13,21,24). Human model, however, showed that the GH concentration in vitreous was lower in diabetic than in nondiabetic patients. This fact rules out the possibility of excessive serum GH passing through the damaged BRB, but indicates autonomous production of GH within the eye (13,25). It is believed that somatostatin (SST), also known as inhibitor of GH secretion, is an important regulator of GH homeostasis in the eye and its production is confirmed in the retinal ganglion cells and retinal pigment epithelial cells (RPE) cells (26,27).

The aim of this study was to explore GH secretion and its dependence on oxidative stress in the eye and also its correlation with GH in the systemic circulation. We assumed that the eye production of GH in oxidative stress was reduced due to the simultaneous apoptosis of neural cells that produce it.

## Patients, material, and methods

This case-control study was conducted at the Zagreb University Clinical Hospital “Sveti Duh,” Opthmalmology Department, Zagreb, Croatia in the period from April 2013 to December 2014. Eligible were patients with previously diagnosed proliferative diabetic retinopathy (PDR) associated with type II diabetes mellitus who underwent classic three port pars plana vitrectomy (PPV). Non-inclusion criteria were positive history of intraocular bleeding or inflammation, history of vitreoretinal surgery or retinal photocoagulation, history of intravitreal steroid or anti-VEGF therapy, current systemic steroid or cytostatic therapy, poorly controlled cardiovascular status, and pregnancy. 32 patients were enrolled. Type II diabetes mellitus was defined according to the World Health Organization criteria, and PDR according to the Early Treatment Diabetic Retinopathy Study criteria. Control group consisted of 49 nondiabetic patients who underwent PPV in the same period for causes other than PDR with no signs of neovscularizations, inflammation, or vascular obstruction. The groups were age and sex matched.

The study was approved by the Ethics Committee of the Clinical Hospital “Sveti Duh”, Zagreb and was conducted at the Ophthalmology Clinic, University Hospital “Sveti Duh”, Zagreb, in accordance with all applicable guidelines: Basics of Good Clinical Practice, the Helsinki Declaration, and the Croatian Healthcare Act and Patients’ Rights Act. All patients gave written informed consent to participate in the study.

### Vitreous and blood sampling

Undiluted vitreous samples (1.0-1.5 mL) were collected at the inception of vitrectomy by aspiration without cutting into a 1 mL syringe attached to the vitreous cutter (Ultravit cutter, Constellation Vision System, Alcon, Johns Creek, GA, USA). The vitreous was obtained under the air infusion before starting intravitreal infusion of a balanced salt solution. Immediately after collection, samples were transferred to a sterile tube with coverlid (Eppendorf, Hamburg, Germany) and taken to the laboratory. Samples were centrifuged at 15 000 g for 10 min at 4°C and separated supernatants were frozen at -80°C until final assessment. Venous blood samples were collected simultaneously and centrifuged at 3500 g for 10 min at 4°C, after which separated serums were frozen at -80°C until they were assayed. In vitreous and serum samples, two oxidative stress parameters, advanced oxidation protein products (AOPP) and lipid peroxidation (LPO), and GH levels were measured. In oxidative stress, major degradation changes occur in cell protein and lipid structures. Therefore, AOPP and LPO were selected for this study among common laboratory tests broadly used for oxidative stress evaluation over last decade.

### AOPP assay

AOPP was assayed with the OxiSelect AOPP ELISA assay kit (Cell Biolabs, Inc., San Diego, CA, USA) according to the manufacturer’s instructions. AOPP content was determined by comparing the tested sample with the chloramine standard curve. Briefly, 200 μL of samples or standards was added to separate wells of the microtiter plate (BIORAD, Hercules, CA, USA). A total of 10 μL of Chloramine Reaction Initiator (part of OxiSelect AOPP ELISA kit) was added. The absorbance of each well was recorded immediately on a spectrophotometric plate reader using a wavelength of 340 nm MRX (Dynex Technologies GmbH, Denkendorf, Germany). Results were calculated according to standard curve and expressed as µM.

### LPO assay

LPO was assayed with Lipid Hydroperoxide ELISA assay kit (Cayman Chemical Company, Ann Arbor, MI, USA) by direct measurement of redox reaction with iron ions according to the kit manual. Briefly, in 500 µL test samples, the solution “LPO Assay Extract R” (part of OxiSelect AOPP ELISA assay kit) was added. To this mixture, 1 mL of cold chloroform solution was added, blended, and centrifuged. A total of 450 µL of mixture chloroform-methanol was added to 500 µL of extract chloroform-sample, followed by a 50 µL mixture of chromogen, which turned purple. The absorbance was measured at 500 nm with a Spectro UVD-3500 spectrophotometer (Labomed Inc, Los Angeles, CA, USA). From the calibration curve of LPO, we calculated the concentration of LPO in each sample according to the formula specified by the manufacturer and expressed as µM.

### GH assay

GH levels were measured by electrochemical luminescence assay using the analyzer Cobas e411 (Roche Diagnostics GmbH, Mannheim, Germany). In the first incubation, sandwich formation of GH samples, byotin labeled monoclonal antibody, and ruthenium labeled specific polyclonal antibody for GH was created. After second incubation with streptavidin, newly formed complex was magnetically bound to electrode surface. Chemical luminescence triggered with controlled voltage and emitted by ruthenium complex was measured with photomultiplicational analyzer. GH levels were calculated from the calibration curve. All the reagents and the calibrator were obtained from the same manufacturer like the analyzer.

### Statistical analysis

Continuous variables are presented as mean ± standard deviation, or as median and range if the distribution was not normal. Normality was assessed using the Kolmogorov-Smirnov test. Categorical variables are presented as percentages. Between group analyses of categorical variables were performed using the *t* test for normally distributed data and Mann-Whitney test for non-normally distributed data. χ^2^ test was used for comparison of categorical data. Correlation was estimated by either the Spearman or Pearson coefficient. All tests were two-tailed and *P* < 0.05 was considered statistically signficant. All analyses were performed using Statistica software package v 12 (StatSoft, Inc., Tulsa, OK, USA, 2014).

## Results

There were no significant differences in sex between control group and PDR group (*P* = 0.829). However, control group was significantly older than PDR group (mean age ± standard deviation, 69.4 ± 11.6 years vs 61.3 ± 12.2 years; *P* = 0.001).

**Figure 1:**
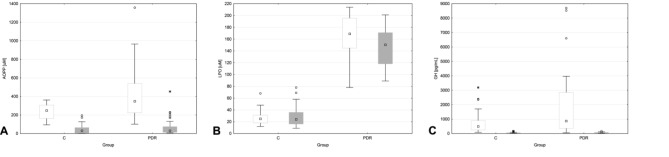
Levels for advanced oxidation protein products (AOPP) (**A**), lipid peroxidation (LPO) (**B**), and growth hormone (GH) (**C**) as median (square), interquartile range (box), non-outlier range (whiskers), outliers (circles), and extremes (asterisks). Levels in serum are shown as white boxes and vitreous levels are shaded in gray. C – control group; PDR – proliferative diabetic retinopathy group.

AOPP levels were significantly higher in serum than in vitreous, both in PDR group (median and range, 348.0 [102.0-1358.0] µM vs 30.50 [6.0-453.0] µM, *P* < 0.001) and control group (248.0 [94.0-362.0] µM vs 30.0 [1.0-195.0] µM, *P* < 0.001) (Figure 1A). PDR group had significantly higher serum AOPP levels than control group (*P* = 0.005), whereas vitreous AOPP levels did not differ significantly between the groups (*P* = 0.179).

**Figure 2:**
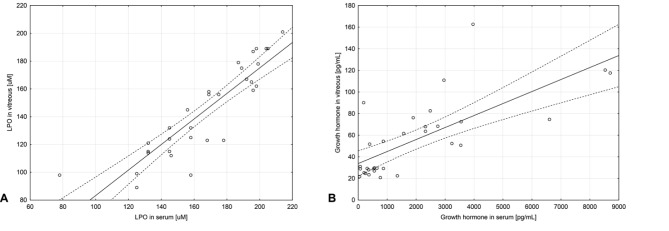
Regression graphs for lipid peroxidation (LPO) levels in serum and vitreous (**A**) and growth hormone levels in serum and vitreous (**B**). C – control group; PDR – proliferative diabetic retinopathy group.

LPO levels were significantly higher in serum than in vitreous in PDR group (169.0 [78.0- 214.0] µM vs 150.5 [89.0-201.0] µM, *P* < 0.001), but not in control group (25.0 [12.0-68.0] µM in serum and 24.0 [9.0-78.0] µM in vitreous, *P* = 0.413). PDR group had significantly higher levels than controls in both serum and vitreous (*P* < 0.001 in both cases) (Figure 1B). Positive correlation between LPO levels in serum and vitreous was detected in both groups. This correlation was not significant in control group (r = 0.228; *P* = 0.115), but was significant in PDR group (r = 0.909; *P* < 0.001). This significant correlation in PDR group was tested for causality using simple linear regression method resulting in high value outcome with R˛ value of 0.780 (*P* < 0.001) (Figure 2).

GH concentrations were significantly higher in serum than in vitreous both in PDR group (866.4 [45.7-8702.0] pg/mL vs 51.3 [20.80-162.7] pg/mL, *P* < 0.001) and control group (492.4 [83.3-3190.0] pg/mL vs 34.0 [20.2-172.4] pg/mL, *P* < 0.001) (Figure 1-C). Serum GH levels were significantly higher in PDR than in control group (*P* = 0.012), while the levels did not differ significantly in vitreous (*P* = 0.439). Similarly to LPO levels, correlation between GH levels in serum and vitreous was significant in PDR group (r = 0.691; *P* < 0.001), but not in control group (r = 0.084; *P* = 0.567). This significant correlation in PDR group was tested for causality using simple linear regression method resulting in high value outcome with R˛ of 0.527 (*P* < 0.001) (Figure 2). We correlated GH levels with AOPP and LPO in serum and vitreous separately, but none of these correlations was significant.

### Discussion

By measuring the concentration of GH and parameters of oxidative stress in serum and vitreous we showed that GH in the eye was independent of serum GH. We also noticed that GH production was not reduced and thus not directly dependent on oxidative stress in the eye of diabetic patients.

Association between GH and DR is well known (9-12,28-30). For decades it was thought that the pituitary GH is one of the main promoters of DR development. However, this role has recently been attributed to IGF1 (15,31). Different medications or surgical and radiological methods were used to influence GH-IGF1 axis with the aim to suppress pituitary gland or circulating growth factors (32-36).

Recent literature indicates that GH is also produced in the eye. The initial investigation was performed on a chicken embryo model, followed by a rat model, and research using bovine model is under way (1,4,37,38). It has been proven that eye GH secretion exists in embryonic life of different animal species even before the start of pituitary gland activity and that its role is important in eye embryogenesis (2,3). In vertebrates and especially mammals, postnatal eye GH activity and its neuroprotective role in postembryonic life have been confirmed (7,8). When suppressed, neuronal deterioration of the optic nerve and some neuronal layers of the retina have been reported (7,8,39,40). These findings have opened new questions about the interaction of GH and DR occurrence.

GH was found in samples of RGC cultures, thus confirming that it is the product of those specific cells and its secretion is assumed to be autonomous and independent of the pituitary (7). Such assumption is based on the fact that the GH secretion in the eye does not decrease with age like in serum and that diabetic patients have lower levels of vitreous GH than non-diabetic participants because of assumed RGC death due to PDR (15,41-43). This is supported by a previous study (31), which also reported reduced vitreous level of IGF1 in diabetic patients since production of IGF1 is GH-dependent. We wanted to investigate the direct relationship between serum and vitreous GH in order to accomplish comparative conclusions. Therefore, we directly compared serum and vitreous samples from the same patients. We used a human model of DR, as was done in previous research (15,16). PDR is a convenient model since it meets two relevant requirements. One is the link between GH and the etiology of DR (10-12) and the other is hypoxia and neural cell death (13,21,25). Our results showed that there were no significant difference between the total GH concentration in diabetics’ and controls’ vitreous. These results are contrary to our expectations based on published data on lower GH levels in diabetic vitreous, which was explained by RGC apoptosis (31,41). It should be noted that our control group had an identical pattern of ocular pathology to that shown in other studies (7,41). However, there are also studies reporting elevated levels of vitreous GH and IGF1 in diabetics (16,44).

Due to the penetration of proteins through the BRB in PDR some authors have additionally corrected vitreous GH concentration for the amount of total protein composition in the vitreous since autonomous GH is free and the rest is bind to the proteins, which made the levels of vitreous GH even lower in absolute terms (15,31,41). Almost identical concentrations of GH in the vitreous of diabetics and controls in our study confirmed independent-secretion of GH in the eye, although the serum GH levels in PDR group were 3-fold higher than the serum levels in control group. This observation also suggests that the BRB, even when it is disrupted like in PDR, actually remains impenetrable for the GH molecule of 22 kDa and thus prevents serum GH to penetrate the eye (45,46). This particular conclusion is in contrast to some observations in animal models, suggesting that disruption of BRB in diabetics allows the passage of molecules up to 70 kDa (24). Since previous studies (15,41) showed decreased levels of GH in diabetic vitreous, explaining them by ruination of RGC layer, equal levels of vitreous GH found in this study remain to be explained.

We assumed that because of the lack of production of intraocular SST in diabetic patients, which is also confirmed in the recent literature, local inhibition of GH is reduced or diminished (47,48). In these patients this should lead to a significant increase in vitreous GH levels, however it does not happen in such an extent, probably due to RGC apoptosis. Finally this leads to roughly equal vitreous GH levels in controls and diabetics, as shown by our results. We assumed that another reason why its level of production remains preserved, despite the decline of ganglion cells, which are the main source of GH, is probably the existence of another GH secretion source in the eye. As a matter of fact, positive findings of GH immunoreactivity were found in the cells of the iris, ciliary, retinal pigment epithelium, lens, cornea, and choroid but this possibly related facts have not been investigated yet (49).

We wanted first to examine quantitative parameters of oxidative stress and then correlate them with GH production to evaluate whether, despite RGC apoptosis, production of GH in the eye can be retained. In diabetics, we found a significant correlation between serum and vitreous for the parameters of oxidative stress and GH, however correlation between oxidative stress and GH production in the eye was not significant. This led us to believe that, in spite of neural cell apoptosis under oxidative stress, GH secretion remains functional, and this fact may confirm the existence of sufficient compensatory GH secretion in other locations of the eye and not only in neural tissues.

Our assumption of non-neural GH secretion in the eye is supported by Ziaei et al (41), who found that the concentration of GH in the vitreous of patients with nonproliferative DR was lower than in patients with proliferative DR. This finding hardly fits the hypothesis that RGC is a major and relevant factor of GH secretion in the eye, especially in conditions of compromised homeostasis and ganglional cell death. Extraneural locations of GH secretion in the eye can obviously compensate to a certain level for a lack of primary neuronal production, which occurs due to neurodegeneration in DR. Moreover, there are certain indicators that suggest that GH may be expressed in any cell (50). An indirect confirmation of the sustained activity of GH is a multiple increase in VEGF in the eye with DR which is a proven fact, and VEGF synthesis requires GH dependent IGF1 (44,47,51).

The question raised in recent years is whether diabetic retinopathy occurs in the exact sequence proposed by the classical basics of biomedical sciences. Vascular changes have long been regarded as the initial change and prominent feature of DR. New research, however, shows that, several years before the appearance of visible vascular changes, processes like excitotoxicity and neurodegeneration are initiated, in which GH produced within the eye plays an important role (47). By comparing the same parameters in serum and vitreous samples, which has not been done so far, this study showed the independence of GH secretion in the eye. It also showed that the production of GH in the eye is not dependent on oxidative stress in DR, most likely due to altered homeostasis of other growth factors and activation of non-neural GH secretion sources inside the eye. We explained some new details of GH behavior in DR and thus contributed to the understanding of new aspects in the development of DR.

Because of the difficulty of collecting large number of vitreous samples, this study is somewhat inferior to the animal studies, but the number of samples tested is very similar to other published human studies (15,31,41,51). It is very difficult to achieve perfect match between the experimental and control groups, but data analysis was still performed as projected in the basic study design.

Further research on the role of hormonal activity of the eye in DR could contribute to the formation of new therapeutic modalities. In particular, it would be interesting to examine the qualitative aspect of GH that is created in oxidative stress, because according to some indications immature growth factors isoforms can be neurodegenerative, and even mature forms may be ineffective or have altered activity due to the hormone receptor modified expression or malfunction (44). Finally, emergence and speed of DR progression varies on an individual basis depending on the DR phenotype (47), which makes further research in this field even more complex. When it comes to therapy, today we already have broad application of VEGF molecules antagonists since their angiogenic role is very pronounced in the course of DR. Similarly, on the basis of new knowledge about the importance of the role of GH, development of new therapeutic options is possible, particularly local application of SST agonists or growth hormone antagonists.
